# Supplementary material for: Transcriptome Analysis and Screening of Genes Associated with Flower Size in Tomato (Solanum lycopersicum)
Source: Int J Mol Sci. 2022 Dec 9;23(24):15624. doi: 10.3390/ijms232415624 (PMC9778759; doi:10.3390/ijms232415624)
Supplement: Supplementary file 1 [file ijms-23-15624-s001.zip › Supplementry files.pdf]

Table S1 Primers used for qRT-PCR

Gene ID	primer-F (5'-3')	primer-R (5'-3')
Solyc04g081000	GACAGAGGATGGGAGAAAGCC	CAGTTGGAAATCCAAGCACGG
Solyc04g015530	CCACCATTGCCACAGGTGAT	ATCCTCCCGGCCAAGTCTTA
Solyc05g056620	TCATGGCATTGTGGTGAGCA	TCAGGCCCTACTTGTGAGGT
Solyc02g087860	AGCAGAAGGTCGAGGCAATC	GATGCCGATGCCTTGCTAGA
Solyc08g067230	GAACTCAGGCACCTAAAGGG	TGTCTGCTCAAACACTTCCCC
Solyc02g071730	CTGCGCGCACAAATTGGAAA	ATGCTGATGCTGAGCTCTCT
Solyc07g062710	TTCTACGCCGTCGGATCAAG	TTCTCCGGGCTCATTCTTCG
Solyc02g083950	TGCAGGCAAAGTAGTAGCCG	AGCAGCAATGAGCCTCTTCTT
SlEF-1α	CCACCAATCTTGTACACATCC	AGACCACCAAGTACTACTGCAC

Table S2 qRT-PCR reaction system

Reactant of the system	The required volume
cDNA	2 μL
primer-F (10 μM)	0.5 μL
primer-R (10 μM)	0.5 μL
2×ChamQ SYBR qPCR Master Mix	10 μL
RNase-Free Water	7 μL
Total volume	20 μL

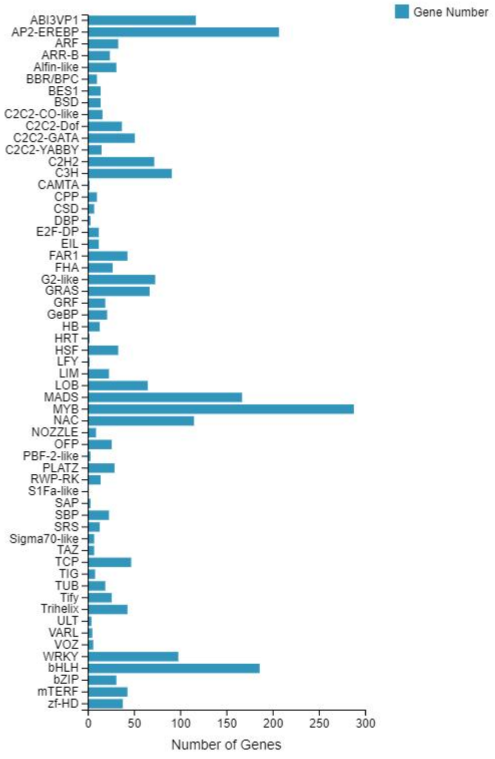

Figure S1. Classification of transcription factor families to which genes belong

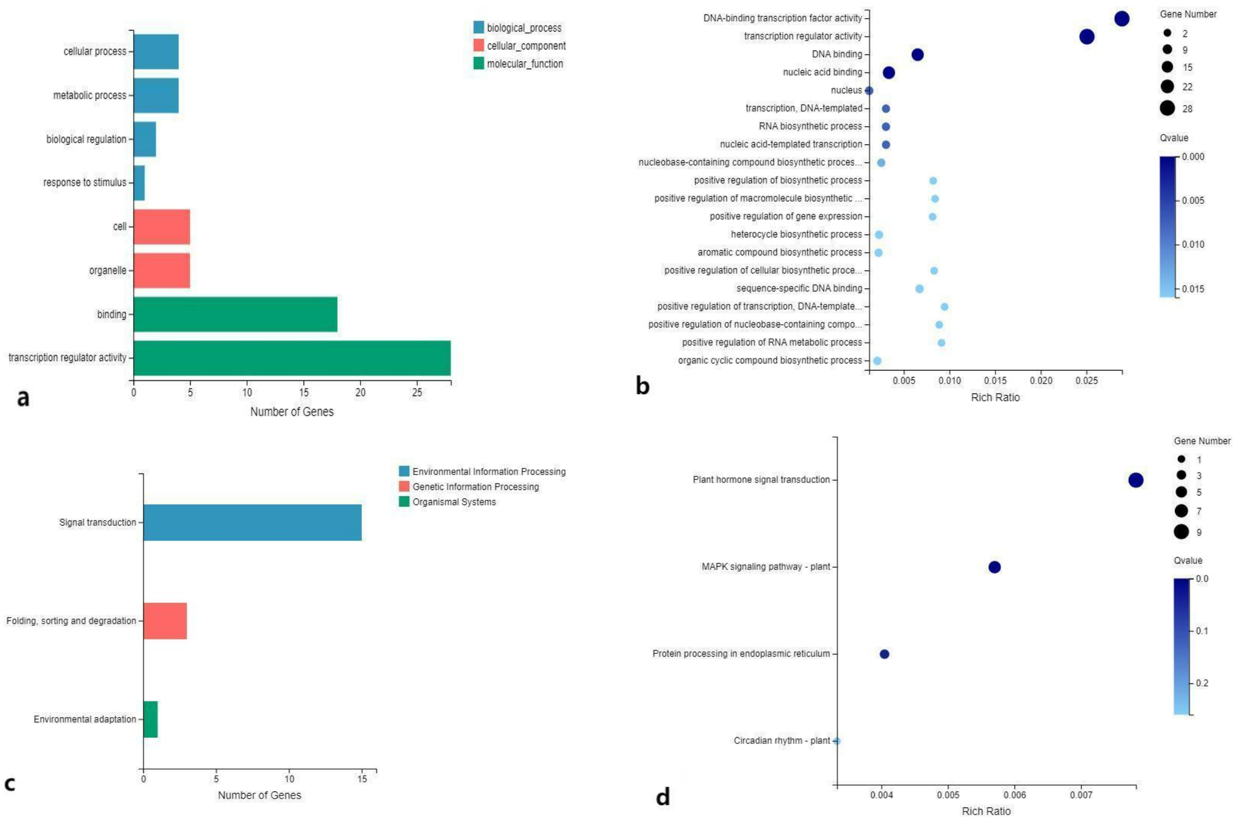

Figure S2.GO analysis and KEGG Pathway analysis of bZIP family.(a)GO classification of bZIP family(b)GO enrichment bubble map of the bZIP family(c)KEGG Pathway of bZIP family(d)KEGG Pathway enrichment bubble map of the bZIP family.

Note: Rich Radio: enrichment ratio, the larger the bubble, the greater the number of genes
